# Distinct lung cancer subtypes associate to distinct drivers of tumor progression

**DOI:** 10.18632/oncotarget.26217

**Published:** 2018-10-30

**Authors:** Valeria Relli, Marco Trerotola, Emanuela Guerra, Saverio Alberti

**Affiliations:** ^1^ Unit of Cancer Pathology, CeSI-MeT, University “G. d’Annunzio”, Chieti, Italy; ^2^ Department of Medical, Oral and Biotechnological Sciences, University “G. d’Annunzio”, Chieti, Italy; ^3^ Department of Biomedical Sciences, Dentistry, Morphological and Functional Imaging, University of Messina, Messina, Italy

**Keywords:** non-small cell lung cancer, lung adenocarcinomas, lung squamous cell carcinomas, prognostic determinants, survival curves

## Abstract

The main non–small-cell lung cancer (NSCLC) histopathological subtypes are lung adenocarcinomas (LUAD) and lung squamous cell carcinomas (LUSC). To identify candidate progression determinants of NSCLC subtypes, we explored the transcriptomic signatures of LUAD versus LUSC. We then investigated the prognostic impact of the identified tumor-associated determinants. This was done utilizing DNA microarray data from 2,437 NSCLC patients. An independent analysis of a case series of 994 NSCLC was conducted by next-generation sequencing, together with gene expression profiling from GEO (https://www.ncbi.nlm.nih.gov/geo/).

This work led us to identify 69 distinct tumor prognostic determinants, which impact on LUAD or LUSC clinical outcome. These included key drivers of tumor growth and cell cycle, transcription factors and metabolic determinants. Such disease determinants appeared vastly different in LUAD versus LUSC, and often had opposite impact on clinical outcome. These findings indicate that distinct tumor progression pathways are at work in the two NSCLC subtypes. Notably, most prognostic determinants would go inappropriately assessed or even undetected when globally investigating unselected NSCLC. Hence, differential consideration for NSCLC subtypes should be taken into account in current clinical evaluation procedures for lung cancer.

## INTRODUCTION

Lung cancer is traditionally classified as non–small-cell lung cancer (NSCLC) and small-cell lung cancer (SCLC) [1]. The two cancer types differ in histopathological traits, genetic changes, prognosis and response to therapy [1]. However, while the usefulness of distinguishing NSCLC from SCLC is clear, far less clear is the reason for jointly categorizing distinct NSCLC subtypes. NSCLC is the most common type of lung cancer, with a poor response to chemotherapy and a low survival rate. This unfavorable treatment response stems from both late diagnosis and from complex, incompletely understood biology. The two main NSCLC histopathological subtypes are lung adenocarcinomas (LUAD) and lung squamous cell carcinomas (LUSC). To define the contribution of major cellular pathways to the biogenesis of LUAD versus LUSC, we profiled their transcriptomic signatures, identified the corresponding control networks and defined key prognostic determinants of biological outcome.

Distinct gene sets have been shown to differentially associate to LUAD versus LUSC. In a study by Charkiewicz et al. [2] a 53 gene signature was identified as diagnostic between LUSC and LUAD [2]. Additional gene sets were identified by Liu et al. [3]. LUAD-associated genes included tight junction and cell adhesion components. Diagnostic assessment revealed p63, TTF1, CK5/6, and Napsin A as efficient diagnostic discriminants for LUAD versus LUSC [4]. Other groups [5, 6] identified gene expression signatures in LUAD patient case series. Overall, though, the studies above did not provide a coherent picture of the tumor progression trajectories of NSCLC subtypes. Furthermore, no high-impact prognostic indicators were consistently identified.

We reasoned that key determinants of tumor identity were likely to be important contributors to the biological history of a tumor. Such determinants would correspondingly impact on the prognosis of distinct NSCLC subtypes. Hence, we went on to explore potential indicators of clinical outcome, by separately analyzing individual determinants associated to distinct NSCLC subtypes. This was done utilizing transcriptomic DNA microarray and associated clinical data from 2,437 NSCLC patients [7]. We examined the impact of gene expression levels in primary tumors on the progression status in NSCLC patients following surgical treatment. Transcriptomic analysis was extended with next-generation sequencing (NGS) of an independent NSCLC case series, to provide technology bias-independent assessment of prognostic mRNA determinants. Expression at the protein level was extensively assessed to validate all the markers utilized in the study.

Our findings led to the discovery of sets of genes that differentially determine disease outcome in distinct subgroups of lung cancers. These included key drivers of tumor growth, which were differentially involved in LUAD versus LUSC development. Our findings provide novel insight into the biological history of LUAD and LUSC and indicate that distinct tumor progression pathways are at work in the two main NSCLC subtypes.

## RESULTS

### Prognostic determinants in LUAD versus LUSC

**Figure 1:**
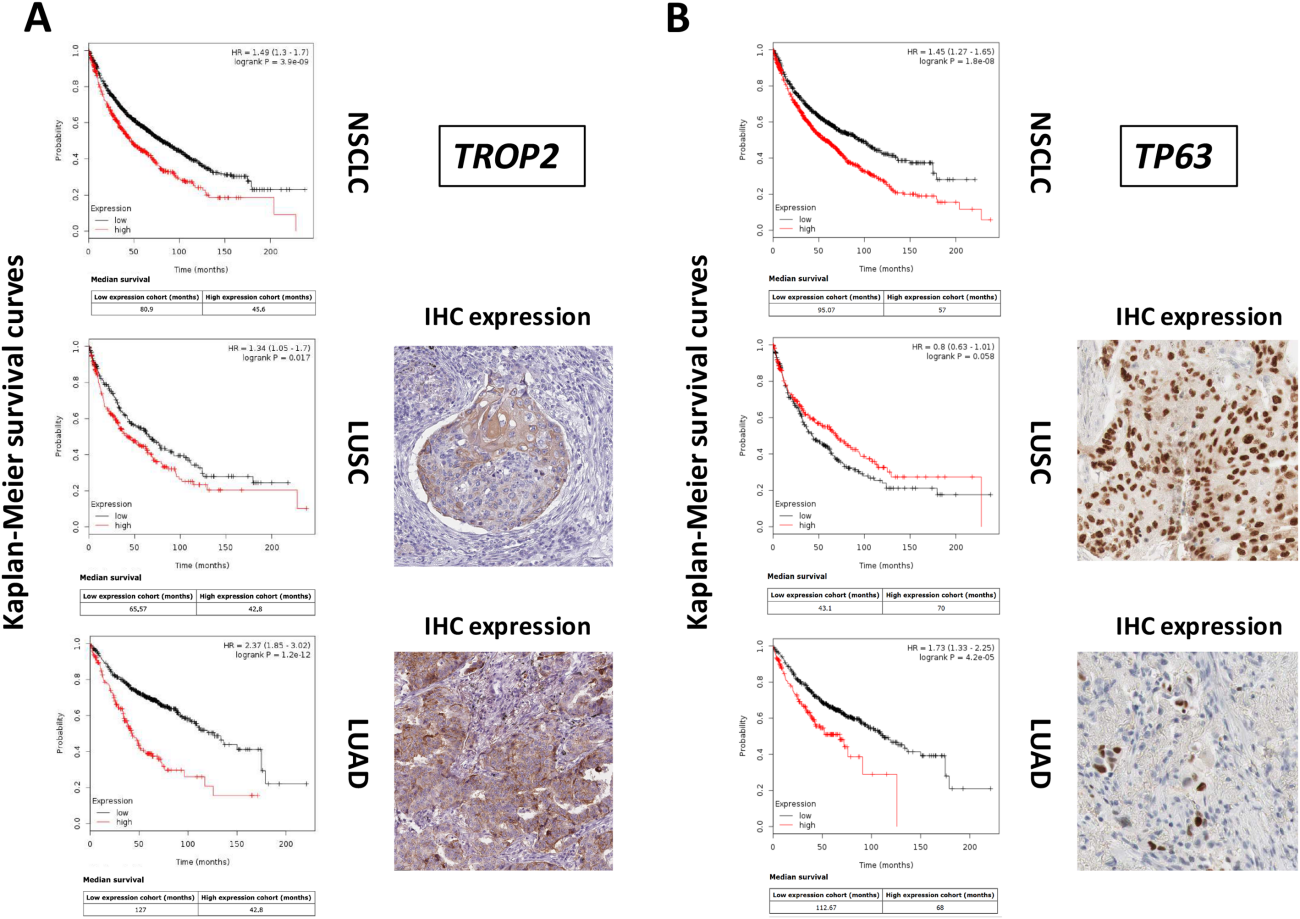
Differential genetic diagnostic and prognostic impact on LUAD versus LUSC DNA microarray data from 2,437 NSCLC patients were preprocessed and meta-analyzed through the KMPlot database (http://www.kmplot.com). Gene expression data were downloaded from GEO (https://www.ncbi.nlm.nih.gov/geo/), using clinical survival information and minimum patient numbers as threshold. Databases containing high-resolution IHC images were analyzed for patterns of expression of differential diagnostic and prognostic proteins for lung LUAD versus LUSC (https://www.proteinatlas.org/). **(A)** KM survival curves of high (red) versus low (black) *TROP2* expressors. Median survival, HR and correlated P values are indicated. **(B)** KM survival curves of high (red) versus low (black) *TP63* expressors. Median survival, HR and correlated P values are indicated. (right side of the panels) IHC analysis of the expression of the Trop-2 or p63 proteins in LUSC or LUAD.

Disease-associated determinants were predicted to bear on clinical outcome. We thus went on to explore the prognostic power of genes that were differentially expressed in LUSC versus LUAD. *TROP2* is a widespread driver of tumor progression [8–10], and was shown to have a negative bearing on unselected cases of NSCLC (Figure 1A). Notably, though, our findings indicated that *TROP2* has a vast negative prognostic impact on LUAD, but only a marginal one on LUSC, where Trop-2 expression associates to terminal differentiation to cornified cells [11], with formation of keratin pearls (Figure 1A). Parallel findings were obtained for *TP63*, which we had previously shown to be both an upstream driver of Trop-2 [8, 9] and a downstream effector [12]. *TP63* is a powerful diagnostic discriminant [13], and a cancer prognostic [14] and predictive [15] factor. Consistent, *TP63* overexpression was shown to have a strong negative impact on LUAD. However, it did associate to a trend for protection in LUSC (Figure 1B, Supplementary Table 1).

These findings raised the issue that LUSC and LUAD may follow profoundly distinct tumor progression trajectories. Hence, we went on to first systematically identify differentially expressed genes in LUAD versus LUSC, through supervised analysis of *in silico* datasets (Table 1, Supplementary Table 1). Then, we assessed such differentially expressed genes for impact on malignant progression of the two NSCLC subtypes [7]. A case series of breast cancer patients [16] was utilized as comparison benchmark.

**Table 1:** Prognostic determinants in LUAD versus LUSC

LUSC prognostic determinants
°LUSC diagnosis^a^
▪Protective factors: DSG3, SERPINB13, MRP5, FOXE1, GRHL3, DLX5 ▪Tumor progression determinants: SFN
°LUAD diagnosis ^b^
▪Protective factors: FOLR1, PLEKHA6 ▪Tumor progression determinants: SFTA3
°Not associated to diagnosis ^c^
▪Protective factors: SKP2, TGFBR2 ▪Tumor progression determinants: MPP5, E2F

LUAD prognostic determinants
°LUSC diagnosis^a^
▪Protective factors: ATP1B3, HPCAL3, SFTA2, COL4A6, MRP5, SLUG, PARD6G, SOX2, CLCA2, RPTPB, JAG1, STF1 ▪Tumor progression determinants: KRT5A, KRT6A, KRT14, KRT17, PERP, SERPINB5, SERPINB13, COL7A1, DSG3, TRIM29, FGFBP1, GLUT1, SFN, TMPRS11D, FOXE1, GRHL3, PTHLH, S100A1, DLX5, ST6GALNAC2
°LUAD diagnosis ^b^
▪Protective factors: TMEM125, TTF1, TASK2, TMC5, ACSL5, FOLR1, RORC, QSOX1, SFTA3, CEACAM6, ATP11A, PLEKHA6 ▪Tumor progression determinants: KRT7, TESC, CLDN3
°Not associated to diagnosis ^c^
▪Protective factors: GSK3B, ATR, SKP2, SMC3, CCND3, PRIM2, IGF1R, TGFBR2, CTNND1, CASK, MPP5, CTGF, PDGFA ▪Tumor progression determinants: PRPF19, MCM4, MCM5, RFC2, PLK1, CDK2, PDGFB, E2F

^a^Utilized for LUSC diagnosis.

^b^Utilized for LUAD diagnosis.

^c^Cancer drivers that are not utilized for differential diagnosis of NSCLC subtypes.

Charkiewicz et al. [2] performed DNA microarray gene expression profiling in a training set of 108 NSCLC samples and a validation cohort of 44 samples [2]. This led to identify a 53 gene signature that efficiently discriminated LUSC from LUAD [2]. Additional gene sets were identified by Liu et al. [3]. Diagnostic trials identified p63, TTF1, CK5/6, and Napsin A as selectively associated to LUAD versus LUSC [4]. Chang and colleagues [6] found a 74-gene signature that discriminated LUAD versus LUSC. Lu and colleagues [5] identified a set of 16 differentially-expressed genes, as involved in the apoptotic execution phase.

**Figure 2:**
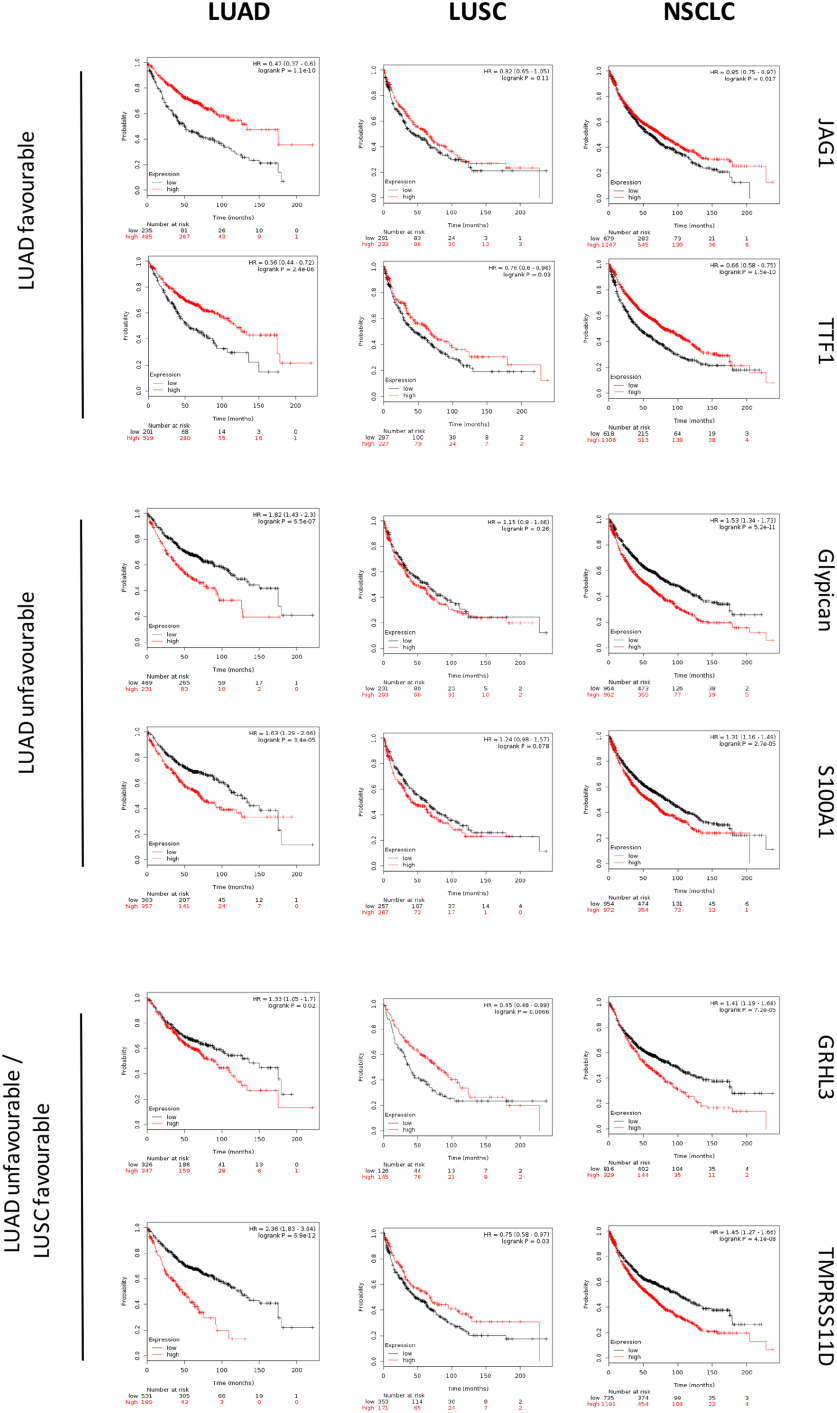
Survival curves of LUAD, LUSC versus unselected NSCLC DNA microarray data from 2,437 NSCLC patients were preprocessed and meta-analyzed through the KMPlot database (http://www.kmplot.com)/GEO (https://www.ncbi.nlm.nih.gov/geo/) as described. KM survival curves of high (red) versus low (black) expressors of the genes indicated on the right are shown. Median survival, HR and correlated P values are indicated. (upper panels) favourable prognostic determinants for LUAD. (mid panels) unfavourable prognostic determinants for LUAD. (lower panels) unfavourable prognostic determinants for LUAD with positive impact on LUSC.

**Figure 3:**
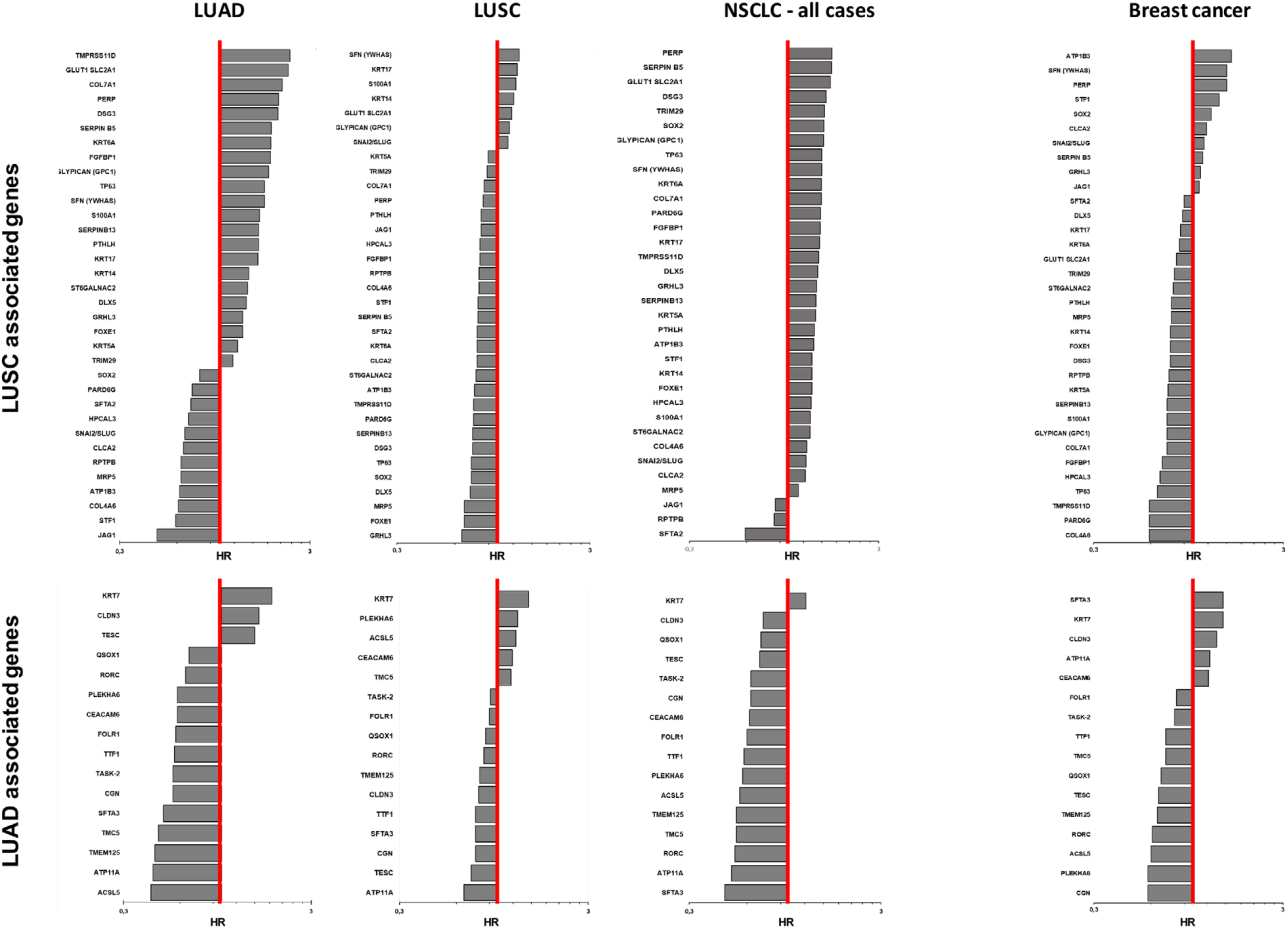
Quantitative impact of prognostic determinants in LUAD, LUSC and breast cancer Bar plots show the hazard ratio (HR)/prognostic impact on overall survival of LUAD, LUSC, NSCLC and breast cancer (http://www.kmplot.com). LUSC-associated genes (upper panel) and LUAD-associated genes (bottom panel) are shown. The genes are listed in descending order of HR values in each tumor type. The red line indicates HR = 1. The bar graphs are plotted on a log scale.

**Figure 4:**
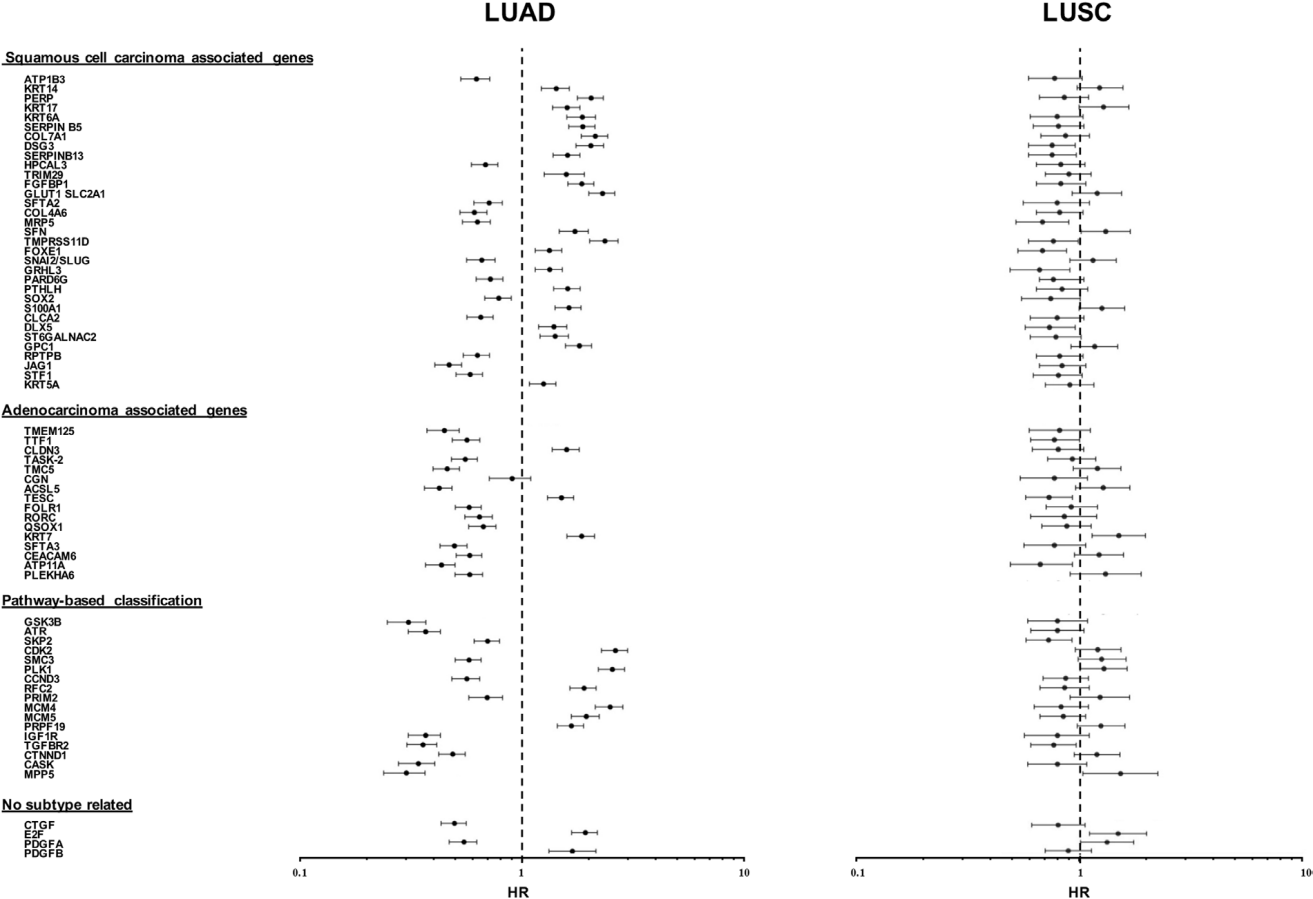
Distribution of disease-outcome values for prognostic LUAD versus LUSC determinants Forest plots summarize the impact of individual determinants overexpressed in LUSC versus LUAD, with opposite prognostic impact in LUAD versus LUSC. The dashed line indicates an HR = 1. Median risk values are indicated by dots. Confidence intervals are indicated by horizontal bars. The graphs are plotted on a log scale. Comparative distribution versus undissected NSCLC is reported in Supplementary Table 1E.

**Figure 5:**
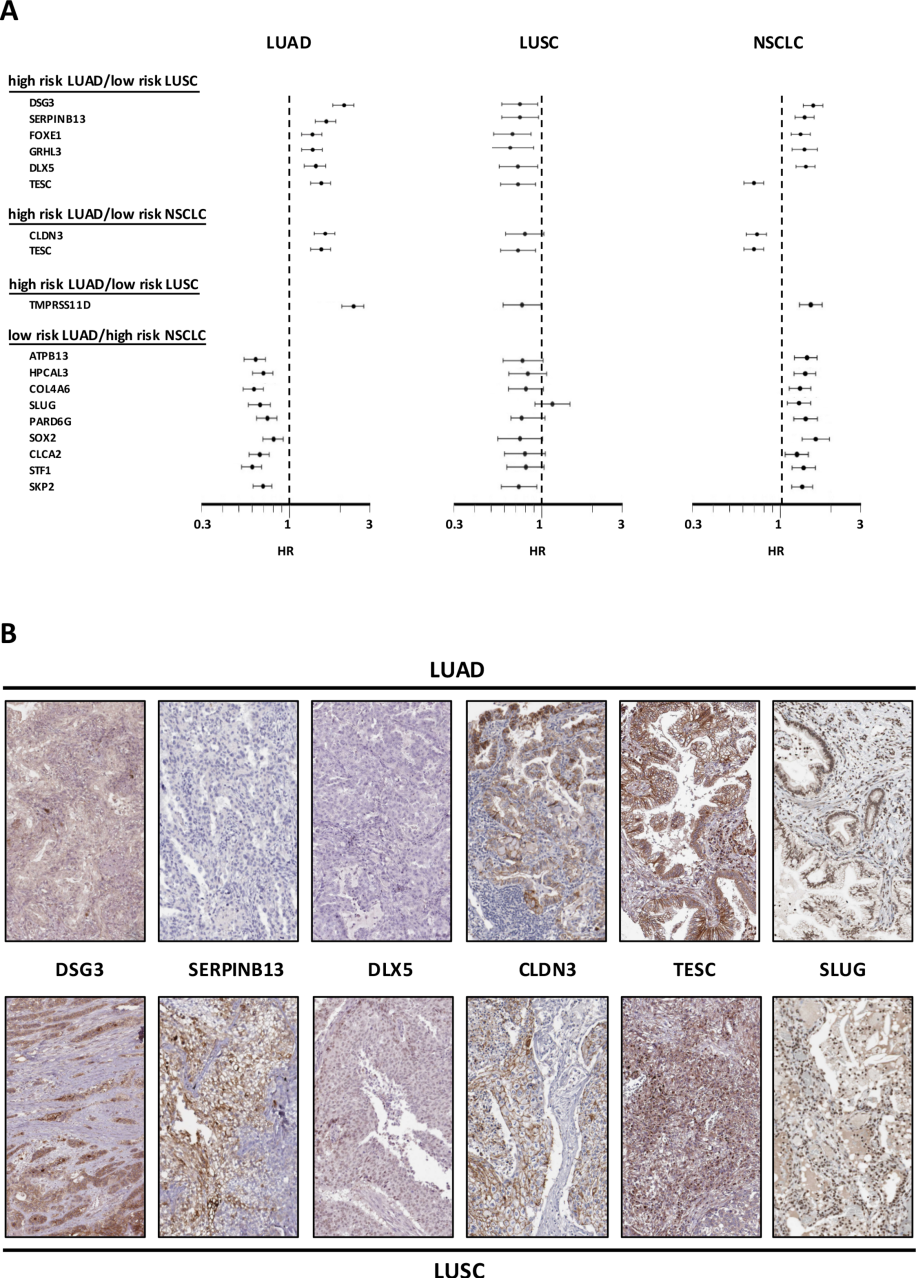
Opposite prognostic determinants in LUAD versus LUSC **(A)** Forest plots of the impact of individual determinants overexpressed in LUSC versus LUAD, with divergent prognostic impact on prognosis of LUAD, LUSC or NSCLC. The dashed line indicates an HR = 1. Median risk values are indicated by dots. Confidence intervals are indicated by horizontal bars. The graphs are plotted on a log scale. **(B)** IHC analysis of the expression of the encoded proteins of representative genes in LUSC or LUAD. Representative examples of average protein expression levels (https://www.proteinatlas.org) are reported.

The sets of differentially expressed genes identified above were parsed for redundancy and validated in silico for differential expression in LUAD versus LUSC. These genes were then systematically assessed for prognostic impact in LUAD versus LUSC, through meta-analysis of DNA microarray data from 2,437 NSCLC patients through the KMPlot database (http://www.kmplot.com). Cox regression analysis was performed and Kaplan–Meier (KM) survival plots were obtained. This led us to identify 69 genes (33 diagnostic for LUSC, 15 associated to LUAD, 21 which were not diagnostic for cancer type), that had significant bearing on prognosis of at least one tumor type (Supplementary Table 1). Remarkably, only 8% of LUSC-diagnostic genes and 21% of LUAD-diagnostic genes showed a concordant impact on lung cancer diagnosis. Strikingly, impact profiles were more similar to benchmark breast cancers (25% of LUAD parameters; 31% of LUSC parameters) than between NSCLC subtypes (Supplementary Table 1, Figures 2-5). Dramatic examples were those of *DSG3, SERPINB13, FOXE1, GRHL3, DLX5*, *TMPRSS11D*, *TESC*, which had a negative prognostic impact on LUAD, but a positive one in LUSC (Figures 4, 5A). Prognostic impact was often obscured when LUAD and LUSC were categorized together as NSCLC, e.g. in the case of *JAG1*, *S100A1*, *KRT7*, *RPTPB*, *CSPG6*, *PDGFB* (Supplementary Table 1). Further, determinants such as *CLND3*, *TESC*, which were high-risk indicators in LUAD, were detected as positive prognostic factors in unselected NSCLC (Figure 5A, Supplementary Table 1), and determinants such as *ATP1B3, HPCAL3, COL4A6, SLUG, PARD6G, SOX2, CLCA2, STF1, SKP2*, which were positive indicators in LUAD, were detected as negative prognostic factors in unselected NSCLC (Figure 5A, Supplementary Table 1).

To validate DNA microarray findings, tumor transcriptomes were profiled by next-generation sequencing (NGS), as an orthogonal technology versus mRNA quantification by hybridization [17]. NGS analysis was conducted on series of 500 LUAD and 494 LUSC cases (https://www.proteinatlas.org/) (Supplementary Table 2). Raw data were obtained for primary assessment, and were computed as scatter plots of individual survival values (Supplementary Table 3).

Comparisons of NGS KM curves versus DNA microarray analysis of corresponding parameters were conducted. Concordance of prognostic impact with significant P values, or correspondence of lack of significant prognostic in specific subgroups were listed. A preliminary quality filter was introduced that eliminated graphs with subgroups of low numerousness (≤100 patients) and cases where the investigated parameter was not detected. Among LUSC diagnostic parameters (96 KM analyses) discordant impact was detected in 14 cases (14.6%). Among LUAD diagnostic parameters (48 KM analyses) discordant impact was detected in 10 cases (20.8%). Among parameters that were not subtype-related (63 KM analyses) discordant impact was detected in 20 cases (31.7 %). Overall, among the 207 KM validated analyses, discordant impact was detected in 44 cases (21.3%) for an overall, highly reliable 78.7% concordance across survival analyses.

### Prognostic genes with the highest impact on disease progression

Genes whereby high versus low expression best discriminated between progressing versus non-progressing tumors encompassed growth factor and growth factor receptors, transcription factors, cell cytoskeleton and cell-cell junction components, together with constituents of the intercellular matrix (Table 1). Overexpression of the transforming growth factor (TGF) receptor 2 gene (*TGFBR2*) was shown to associate with less aggressive disease courses in LUSC. Correspondingly protective determinants for LUAD were *IGF1R* and *PDGFA*, whereas *PDGFB* had a negative prognostic impact on LUAD. Keratins are used as IHC markers in clinical diagnostic assays, e.g. keratin 5 and 6 for LUSC diagnosis, keratin 7 for LUAD identification. Notably, though, all overexpressed keratins, i.e. *KRT5A, KRT6A, KRT7, KRT14, KRT17*, were found to play a role as LUAD tumor progression determinants. The collagen gene *COL4A6* was shown to be a LUAD protective factor, whereas *COL7A1* associated to worse prognosis. *SERPINB5*/maspin and *SERPINB13* overexpression associated to worse disease outcome in LUAD, but showed a protective role in LUSC.

### Pathway regulation by prognostic genes in lung cancer

The functional role of the identified prognostic genes was assessed versus categorized gene expression data from GEO (https://www.ncbi.nlm.nih.gov/geo/). Main pathways were found to be regulation of the cell cycle (*GSK3B, ATR, SKP2, CDK1, CDK2, CDK4, SMC3, PLK1, CCND3*), control of DNA replication (*RFC2, PRIM2, MCM4, MCM5*) and DNA repair (*ATR*). Cell differentiation appeared also involved, as the transcription factors TTF1, a main LUAD protective factor, is a key player in lung epithelium development. *QSOX1, SOX2*, *SLUG, STF1* were all associated to a more benign course of disease, suggesting control of tumor initiation and cancer stem-cell functions versus epithelial-mesenchimal transition in NSCLC. *SOX2*, and *TP63* are often coamplified and their overexpression is associated to favourable disease outcome (Figures 1B, 3, 4) [18]. Cell-cell adherent junction components, such as *CTNND1, CASK, MPP5*, were associated to a better prognosis of LUAD, likely because of retained cell-cell junctions and epithelial differentiation. High levels of the transmembrane protease *TMPRSS11D* were previously shown to predict poor overall survival in NSCLC [19]. Our findings show that this is entirely due to the dismal outcome of expressing LUAD, as *TMPRSS11D* is associated to favourable prognosis in LUSC (Supplementary Table 1).

### Network analysis

**Figure 6:**
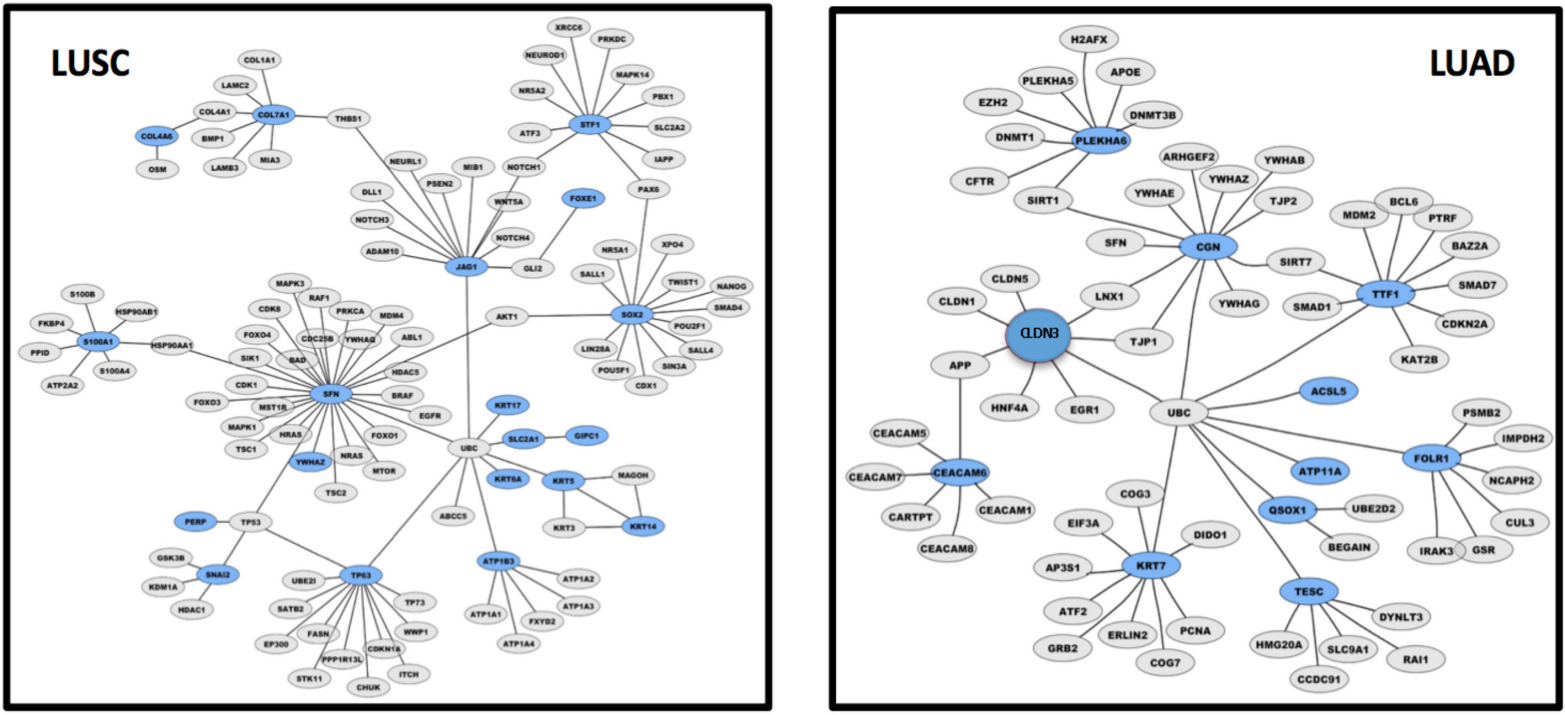
LUAD versus LUSC control gene networks Graphical representation of control gene networks, as identified with Cytoscape 3.6.0. Genes are represented as nodes, biological relationships between nodes are represented as lines. Genes overexpressed in LUSC (left panel) and in LUAD (right panel) are highlighted in blue; direct interactors are in gray.

Whole transcriptome profiling of LUAD versus LUSC and differential prognostic analysis were utilized to reveal potential malignant progression-inducing modules, for intra-group and inter-group differentiation. Control networks involved in cell growth and apoptosis were shown to be profoundly different in LUAD versus LUSC [3, 6, 8, 20, 21] (Figure 6). A pivotal p53/p63/p73 axis only emerged in LUSC (Figure 6, left panel) [20]. Additionally different molecular networks between lung LUAD and LUSC were found in the control of cell cycle, DNA repair, and metabolic pathways.

### Proteomic signature of the differential prognostic profiles

Analysis conducted at the RNA level were extended to the protein level (Supplementary Table 4). *In silico* IHC analysis was conducted, for expression of the encoded proteins in independent cases of patients with lung cancer (Supplementary Table 4). Antibody staining validation included immunofluorescence, Western blotting, reactivity against recombinant protein and comparison of staining patterns of independently-generated antibodies (https://www.proteinatlas.org/about/assays+annotation#iha). All IHC images were checked for staining intensity and fraction of stained cells. Consistent, proteins encoded by genes preferentially associated to LUAD versus LUSC were found preferentially overexpressed by the corresponding tumor types. Only a few exceptions were found, consistent with an additional layer of regulation at the protein translation level. Keratin 17 was indeed found frequently overexpressed also in LUAD, thus questioning its diagnostic role in distinguishing between the two tumor types. Corresponding findings were obtained for JAG1. FGFBP1 and ST6GALN2 proteins were found to be weakly expressed in lung cancers irrespective of tumor type.

## DISCUSSION

Lung cancer is the most common cause of cancer death worldwide, with an estimated 1.6 million deaths each year [22, 23]. NSCLC are often diagnosed at an advanced stage, and the overall 5-year survival for these patients is only 15%–20%. Particular histotypes of NSCLC may display distinct molecular characteristics and molecular determinants, which may associate with distinct histopathological and genetic characteristics of lung cancer [6]. However, current clinico-pathological staging procedures appear profoundly inadequate to dissect NSCLC into patient groups with distinct biological outcome.

Transcriptome analysis has the potential to discriminate between distinct tumor types [1]. Transcriptome analysis of independent cohorts of NSCLC cases by Charkiewicz et al. [2] led to the identification of 53 genes, that were differentially expressed in LUSC versus LUAD. Additional gene-sets were identified by Liu et al. [3]. These differentially expressed genes appeared involved in pathways related to cell proliferation, signal transduction and metabolism. Diagnostic trials identified p63, TTF1, CK5/6, and Napsin A as selectively associated to LUAD versus LUSC [4]. Chang and colleagues [6] found a 21-gene signature in the HMGB1/RAGE signaling pathway, 22 risk-modulatory genes of the ERK pathway, as triggered by beta-adrenergic receptors, and a 31-gene signature as associated to clathrin-coated vesicle recycling. Lu and colleagues [5] identified gene expression signatures in 700 LUAD cases. Among them, a set of 16 genes appeared involved in the control of the apoptotic execution phase. Transcriptional network analysis showed involvement of E2F, CTGF, and PDGF in lung cancer pathogenesis [21]. Additionally, LUSC show involvement of the EGF, IL1F8, and CX3CL1 pathways, while changes in Rb1, miR-200, and EMP2 targets appear specific for LUAD [21].

Despite these efforts, little consistency was found across independent studies [6]. Inadequate study design, small sample size and varied data analysis strategies negatively influenced study outcomes. An epitomic analysis of 47 published gene expression signatures, indicated that the predictive performance of several signatures was not superior to that obtained from random gene expression signatures, and sometimes performed even worse [6]. Rather unsettling, over 90% of random signatures with more than 100 gene members appeared to bear a value as significant predictors of disease outcome [6]. Not surprisingly, to date, the only molecular traits that are of current use in clinical practice for prognostic and therapeutic purposes are genomic mutations of EGFR and KRAS and gene fusions of ALK.

We speculated that a main structural weakness of previous approaches was a blunt, overall assessment of NSCLC, as if they were a homogeneous tumor type. Categorization of divergently associated determinants was thus expected to be blurred, if not entirely lost, in such joint classification of NSCLC. Further, association to specific tumor histotypes is expected to stem from at least two distinct and opposing mechanisms. One is positive selective pressure for increased fitness and/or proliferative capacity of expressing cells. A second one is retention of differentiation traits. As such, the first mechanism is expected to be linked to malignancy, the second one, an example is Trop-2 expression terminally differentiated cornified cells in LUSC, is expected to be linked to more benign outcomes. These and additional findings supported the idea of reassessing NSCLC in a dichotomic scenario.

We thus decided to explore potential indicators of clinical outcome, by separately assessing individual determinants versus association to distinct NSCLC subtypes. This was done utilizing DNA microarray data from 2,437 NSCLC patients [7] and 3,951 control breast cancers [16]. We examined the impact of gene expression levels in primary tumors on the progression status in NSCLC patients following surgical treatment. Distinct technologies (microarray versus next-generation sequencing versus real-time polymerase chain reaction), bear distinct limitations and detection bias, such as different probe designs and signal detection methods, or hybridization bias and thermodynamic limitations for DNA microarrays [17]. Hence, transcriptomic analysis was independently conducted through DNA microarray and NGS analysis. Large data set size, quality selection and modular cut-off values were utilized. This led us to obtain the key finding of 78.7% concordance between the two technologies. Further validation for such analyses was provided by confirmation of coherent protein expression of the analyzed genes.

Analysis for differential impact on clinical outcome then led us to discover sets of genes that differentially determine disease outcome in lung LUAD versus LUSC. These included growth factor and growth factor receptors, transcription factors, cell cytoskeleton and cell-cell junction components, together with constituents of the intercellular matrix. Overall, 69 genes were identified, that acted as prognostic determinants. Remarkably, these only had a concordant impact in 8% of LUSC-diagnostic genes, and in 21% of LUAD-diagnostic genes. Several determinants were shown to have a negative prognostic impact on LUAD, but a positive one in LUSC, such as *DSG3, SERPINB13, FOXE1, GRHL3, DLX5, TMPRSS11D*. High-risk indicators in LUAD, were detected as positive prognostic factors in unselected NSCLC, e.g. *CLND3, TESC*. Low-risk indicators in LUAD, such as *ATP1B3, HPCAL3, COL4A6, SLUG, PARD6G, SOX2, CLCA2, STF1, SKP2*, were detected as negative prognostic factors in unselected NSCLC. In several other cases, prognostic impacts were simply obscured when LUAD and LUSC were categorized together as NSCLC, and were lost to further prognostic analysis.

An additional result of our analysis was to help shedding light on driving signaling paths for lung cancer development. Keratin intermediate filaments play a structural role in cornified epithelia and in epidermis development [18]. Notably, all overexpressed keratins, i.e. KRT5A, KRT6A, KRT7, KRT14, KRT17, were found to play a role as LUAD tumor progression determinants. Experimental evidence had previously indicated that keratin 19 (CYFRA21-1) had a negative prognostic impact on LUAD only [24]. Thus, inappropriate expression of keratins appears as a general trait associated to LUAD aggressiveness, whether through perturbation of the differentiation status of adenocarcinoma cells or by regulation of epithelial progenitor/stem cells, as recently shown for keratin 14 [18]. The collagen gene *COL4A6* was shown to be a LUAD protective factor, as possibly related to its regulatory role on cytokeratin expression and epithelial differentiation [25]. *COL7A1* acted as a tumor progression determinant, possibly through its association to cancer stem cells development [26]. *SERPINB5*/maspin is a putative tumor suppressors, through influence on cell-matrix interactions [27]. However, *SERPINB5* disregulation occurs early during multi-step progression models of ductal pancreatic adenocarcinomas, and its overexpression associated to dismal prognosis [28], as we found in LUAD. As in LUSC, downregulation of *SERPINB13* expression in head and neck squamous cell carcinomas was shown to associate with a poor differentiation grade of the tumors, presence of lymph node metastases and a decreased disease-free and overall survival [29].

Taken together, our findings thus indicate that distinct tumor progression pathways are at work in LUAD and LUSC NSCLC subtypes, and that specific determinants have a distinct impact on patient outcome, depending on tumor histology. As such, they should be taken into account in current clinical settings. This separate classification framework may correspondingly help developing and assessing novel diagnostic, prognostic and therapeutic procedures for lung cancer.

## MATERIALS AND METHODS

### Patient case series

For DNA microarray studies, correlated clinical and pathological data were obtained from 2,437 patients with NSCLC [7] and from 3,951 control breast cancers [16] from the Kmplot database (http://www.kmplot.com). Samples from The Cancer Genome Atlas (TCGA) repositories (https://cancergenome.nih.gov/) were parsed versus published gene expression data and survival information. Additional data were obtained from the National Cancer Informatics Program (NCIP) (https://cbiit.cancer.gov/ncip/ncip-home) and the Gene Expression Omnibus (GEO) (http://www.ncbi.nlm.nih.gov/geo/). For analysis of overall survival of NSCLC subgroups, lung cancer datasets were analyzed that were selected using the keywords “lung”, “cancer”, and related ones, and that included microarray gene expression data and correlated clinical characteristics including survival. To test for randomness, pairwise rank tests were performed for the collected clinical data, which included age, sex, smoking history, histology, stage, grade, surgery, radiotherapy and chemotherapy. For breast cancer, reference databases were established using gene expression data and survival information from 3,951 patients [16]. The median relapse-free survival was 6.43 years, 78% patients were estrogen receptor positive, and 14% were lymph node positive. In all cases, for quantification of impact on outcome for each tested parameter, cohorts were divided into two groups, i.e. high versus low expressors, by auto selecting the best cutoff of gene expression by DNA microarray analysis. The analysis was run on selected probe set for each gene, as listed in Supplementary Table 1. Patients surviving over the selected threshold were censored instead of being excluded. In the case of breast cancer the analysis was conducted without stratification by molecular subtype and therapy [16].

NGS analysis for NSCLC was performed on TCGA data from 994 patients (494 LUSC, 500 LUAD). The NSCLC dataset included 398 females and 596 males. Most patients (N = 600) were still alive at the time of data collection. The stage distribution was 510 stage I patients, 277 stage II patients, 163 stage III patients, 32 stage IV patients. Stage information was missing for 12 patients. NGS analysis forbreast cancer was performed on TCGA data from 1075 patients. The dataset included 1063 females and 12 males. Most patients (N = 923) were still alive at the time of data collection. The case series included 180 stage I patients, 609 stage II patients, 243 stage III patients, 20 stage IV patients. Stage information was missing for 11 patients.

### DNA microarray meta-analysis

DNA microarray data from the NSCLC and breast cancer patients were preprocessed and meta-analyzed through the KMPlot database (http://www.kmplot.com). Only Affymetrix HG-U133A (GPL96) and HG-U133 Plus 2.0 (GPL570) microarrays were considered, as they share 22,277 probe sets, to minimize variation in precision, different relative scales, and different dynamic ranges. Oligonucleotide probes for DNA array analysis were chosen for optimal hybridization to Affimetrix chips and highest signal-to-noise ratio between experimental groups. Final data were obtained through 54,675 Affymetrix probe set IDs and 70,632 gene symbols. The raw CEL files were MAS5 normalized in the R statistical environment using the Affymetrix Bioconductor library. Cox regression analysis, KM survival plots, hazard ratios (HR) with 95% confidence intervals, survival scatter plots and logrank P values were computed.

### NGS transcriptome profiling

NGS transcriptomes were profiled utilizing correlated TCGA data from 994 samples from patients with lung cancer (500 LUAD; 494 LUSC) and 1075 patients with breast cancer. The TCGA RNA-seq data was mapped using the Ensembl gene id available from TCGA, and the FPKMs (number Fragments Per Kilobase of exon per Million reads) for each gene were subsequently used for quantification of expression with a detection threshold of 1 FPKM.

KM curves were obtained from analysis of correlation between mRNA expression level and patient survival. Genes with a median expression value lower than FPKM 1 were excluded. The prognosis of each group of patients was examined by KM survival estimators, and survival outcomes were compared by log-rank tests. Genes with log rank P values <0.001 in maximally separated KM analysis were classified as having prognostic impact.

### Immunohistochemistry

Five μm sections from tumor samples were mounted on silanized slides. Tissue peroxidase activity was blocked with 3% H_2_O_2_ for 5 minutes. Slides were quenched with 0.3% BSA in Tris-buffered saline, at room temperature, for 30 min. For Trop-2 staining, antigen retrieval was performed by microwave treatment at 750 W for 10 min using 10 mM sodium citrate buffer pH 6.0 or 1 M urea buffer pH 8.0 (Dako), respectively. Slides were then incubated at room temperature for 30 min with the relevant antibodies. Anti-mouse (K4001, EnVision kit, Dako) and anti-goat (K0679, LSAB kit, Dako) secondary kits were used for signal amplification, as appropriate. Control sections were treated with isotype-matched immunoglobulins or non-immune serum. Slides were washed in Tris-buffered saline-Tween 20, and incubated for 10 min in 3,3’-diaminobenzidine (DAKO). Counterstaining was performed with hematoxylin. Slides were mounted with Immunomount (Shandon). Trop-2 expression was quantified as percentage of stained cells and as intensity of the staining. An IHC score was then obtained, ranging from 0 to 12 [30]. Trop-2 expression levels were analyzed with a goat anti-Trop-2 polyclonal antibody (AF650, R&D Systems). Antigen retrieval was performed by microwave treatment at 750 W for 10 min in 1 M urea buffer (pH 8.0). The LSAB kit (K0679, Dako) was used for signal amplification. Trop-2 antigen expression was scored positive in presence of a specific staining on the tumor cell membrane, and was quantified as percentage of stained cells and as intensity of the staining. The immunostaining score (H-score) was also determined according to the following 5 categories: 0 (0% of positive cells), 1 (<10 % of positive cells), 2 (10–50% of positive cells), 3 (50–80% of positive cells), 4 (>80% of positive cells). The intensity score represented the average intensity of the positive cells as follows: 1 (weak staining), 2 (moderate staining) and 3 (strong staining). The proportion and intensity scores were then multiplied to obtain the H-score, which could range from 0 to 12. To perform the crosstab analysis (chi-square test) between Trop-2 expression and clinicopatholigical features of patients, the protein H-score was dichotomize using a cut-off > 4 [30].

Databases containing high-resolution IHC images were analyzed for patterns of expression of differential diagnostic and prognostic proteins for lung LUAD versus LUSC. The Human Protein Atlas (v. 12, https://www.proteinatlas.org/) provides spatial distribution and expression data from 16,621 proteins/21,984 antibodies and corresponding mRNA in normal human tissues and different cancer types. The expression profiles of distinctly expressed proteins in lung cancer were generated for antibody staining parameters, intensity, and fraction of positive cells in normal cells and cancers originating from different tissues [10].

### Gene ontology, networks, and functional impact

Gene Ontology analysis was performed using PANTHER 7.2 software. The signaling hubs and connectivity networks were obtained using NetworkAnalyst. To condense the first-order network to its major components, a “minimum interaction network” was generated using the “Trim” function as indicated.

### Statistical analysis

Statistical analysis was performed using GraphPad software (https://www.graphpad.com/). Disease free survival (DFS) was defined as the time from surgery to tumor recurrence at local or distant sites. Local relapse free survival (LRFS) and distant relapse-free survival (DRFS) were defined accordingly. KM plots were used to illustrate the survival in specified cohorts. Log-rank tests assessed equality of survival curves. SPSS software Version 15.0 was used throughout these analyses. All P-values were two-sided.

## SUPPLEMENTARY MATERIALS TABLES











## Abbreviations

DFS: disease free survival
DRFS: distant relapse-free survival
FPKM: number fragments per kilobase of exon per million reads
HR: hazard ratios
KM: Kaplan–Meier
LRFS: local relapse free survival
LUAD: lung adenocarcinoma
LUSC: lung squamous cell carcinoma
NGS: next-generation sequencing
NSCLC: non–small-cell lung cancer
SCLC: small-cell lung cancer
TCGA: The Cancer Genome Atlas
