# Supplementary material for: Distinct lung cancer subtypes associate to distinct drivers of tumor progression
Source: Oncotarget. 2018 Oct 30;9(85):35528–40. doi: 10.18632/oncotarget.26217 (PMC6238974; doi:10.18632/oncotarget.26217)
Supplement: Supplementary file 1 [file oncotarget-09-35528-s001.pdf]

Distinct lung cancer subtypes associate to distinct drivers of tumor progression

SUPPLEMENTARY MATERIALS

Supplementary Table 1A: NSCLC and breast cancer prognostic determination through the KMPlot database - Squamous cell carcinoma associated genes

See Supplementary File 1

Supplementary Table 1B: NSCLC and breast cancer prognostic determination through the KMPlot database - Adenocarcinoma associated genes

See Supplementary File 1

Supplementary Table 1C: NSCLC and breast cancer prognostic determination through the KMPlot database - Pathway-based classification §

See Supplementary File 1

Supplementary Table 1D: NSCLC and breast cancer prognostic determination through the KMPlot database - no subtype related§

See Supplementary File 1

Supplementary Table 2A: NSCLC and breast cancer prognostic determination obtained by TCGA RNA-seq data - Squamous cell carcinoma associated genes

See Supplementary File 2

Supplementary Table 2B: NSCLC and breast cancer prognostic determination obtained by TCGA RNA-seq data - Adenocarcinoma associated genes

See Supplementary File 2

Supplementary Table 2C: NSCLC and breast cancer prognostic determination obtained by TCGA RNA-seq data - Pathway-based classification [§]

See Supplementary File 2

Supplementary Table 2D: NSCLC and breast cancer prognostic determination obtained by TCGA RNA-seq data- no subtype related[§]

See Supplementary File 2

Supplementary Table 3A: NSCLC and breast cancer prognostic determination - Scatter plots - Squamous cell carcinoma associated genes

See Supplementary File 3

Supplementary Table 3B: NSCLC and breast cancer prognostic determination - Scatter plots - Adenocarcinoma associated genes

See Supplementary File 3

Supplementary Table 3C: NSCLC and breast cancer prognostic determination - Scatter plots - Pathway-based classification [§]

See Supplementary File 3

Supplementary Table 3D: NSCLC and breast cancer prognostic determination - Scatter plots - no subtype related[§]

See Supplementary File 3

Supplementary Table 4A: NSCLC and breast cancer prognostic determination - IHC analysis - Squamous cell carcinoma associated genes

See Supplementary File 4

Supplementary Table 4B: NSCLC and breast cancer prognostic determination - IHC analysis - Adenocarcinoma associated genes

See Supplementary File 4

Supplementary Table 4C: NSCLC and breast cancer prognostic determination - IHC analysis - Pathway-based classification [§]

See Supplementary File 4

Supplementary Table 4D: NSCLC and breast cancer prognostic determination - IHC analysis - no subtype related[§]

See Supplementary File 4
